# Quantum sensing for NASA science missions

**DOI:** 10.1140/epjqt/s40507-025-00360-3

**Published:** 2025-05-21

**Authors:** Carolyn R. Mercer, Erica N. Montbach, Steven D. Christe, Robert M. Connerton, Denise A. Podolski, Michael P. Robinson, Mario R. Perez

**Affiliations:** 1 NASA Headquarters, Washington, USA; 2 NASA Glenn Research Center, Cleveland, USA; 3 NASA Goddard Space Flight Center, Greenbelt, USA

**Keywords:** Quantum sensing, Remote sensing, Spacecraft, Space science, Earth science

## Abstract

The National Aeronautics and Space Administration (NASA) develops a broad range of technologies to support space-based quantum sensing and communications, uses the space environment to study fundamental quantum processes to advance our knowledge of physics, and develops algorithms to attack complex science problems that might be solved using quantum computing. This paper describes quantum sensors that NASA has flown on space missions, investments that NASA is making to develop quantum sensors, and possible approaches to employ quantum sensing to study the attributes of distant stars and planets, the Sun, Earth, and fundamental properties of matter.

## Introduction

NASA develops technologies to ensure that there is a pipeline of innovation for new space-based missions that will enable us to uncover the secrets of the universe, protect life in space and on Earth, and understand if we are alone in the universe. We strive to deploy science instruments that are better than we’ve used before or are applied in new ways. While “better” can mean lower size, weight, power, or cost, it often means improved spatial, temporal, spectral, and/or energy resolution; incorporation of new features such as absolute calibration; or the ability to make measurements that were previously impossible. Quantum technologies offer the promise to realize these types of improvements and dramatically increase our ability to study the attributes of distant stars and planets, the Sun and Earth, and fundamental properties of matter.

Quantum sensors have existed for decades, with many now available commercially. These sensors are based on a variety of quantum properties, but all enable very precise measurements or novel measurement capabilities. Quantum sensing techniques include the ultra-precise measurement of quantized packets of energy, the manipulation of quantized atomic states to measure macroscopic properties, exploiting the quantum transition to superconductivity, quantum tunneling, and the manipulation of “Cooper pairs” (i.e., paired entangled electrons). Newer techniques include the use of atom interferometry, quantum squeezed light, and quantum entanglement.

NASA’s use of quantum sensing began at the dawn of the space age and continues through the 2023 launch of the X-Ray Imaging and Spectroscopy Mission (XRISM) and beyond. NASA is investing in a broad range of new technologies to enable their use on spacecraft. This effort requires robust instruments that can not only withstand launch loads and the space environment, but also employ practical limits on size, weight, and power usage. Note that in addition to imposing constraints, the space environment, yields benefits; for example, the absence of gravity enables colder temperatures and much longer coherence times for weakly trapped atoms or atoms in free fall, which allows longer interrogation times and therefore higher signal-to-noise ratios.

This paper describes quantum sensing technology that has been deployed by NASA, technology that is being developed by NASA, and future approaches that NASA may take for quantum sensing. Quantum sensors that have been flown on missions or used in ground support are described in Sect. 2, with brief descriptions of the technology and explanations of the application. Section 3 describes NASA’s current investments in quantum sensing technology. Both sections are arranged by the expected space science discipline for application. Finally, a brief description of potential future quantum sensing approaches is provided in Sect. 4, including ideas that have been generated from around the world. Much of the work described in this paper relies on a deep heritage of development throughout the science community. Because this is a review paper, the number of references is purposefully limited and no attempt is made to describe the richness of research that is underway elsewhere or that preceded this work.

## Deployed systems

### Magnetometry

**Figure 1:**
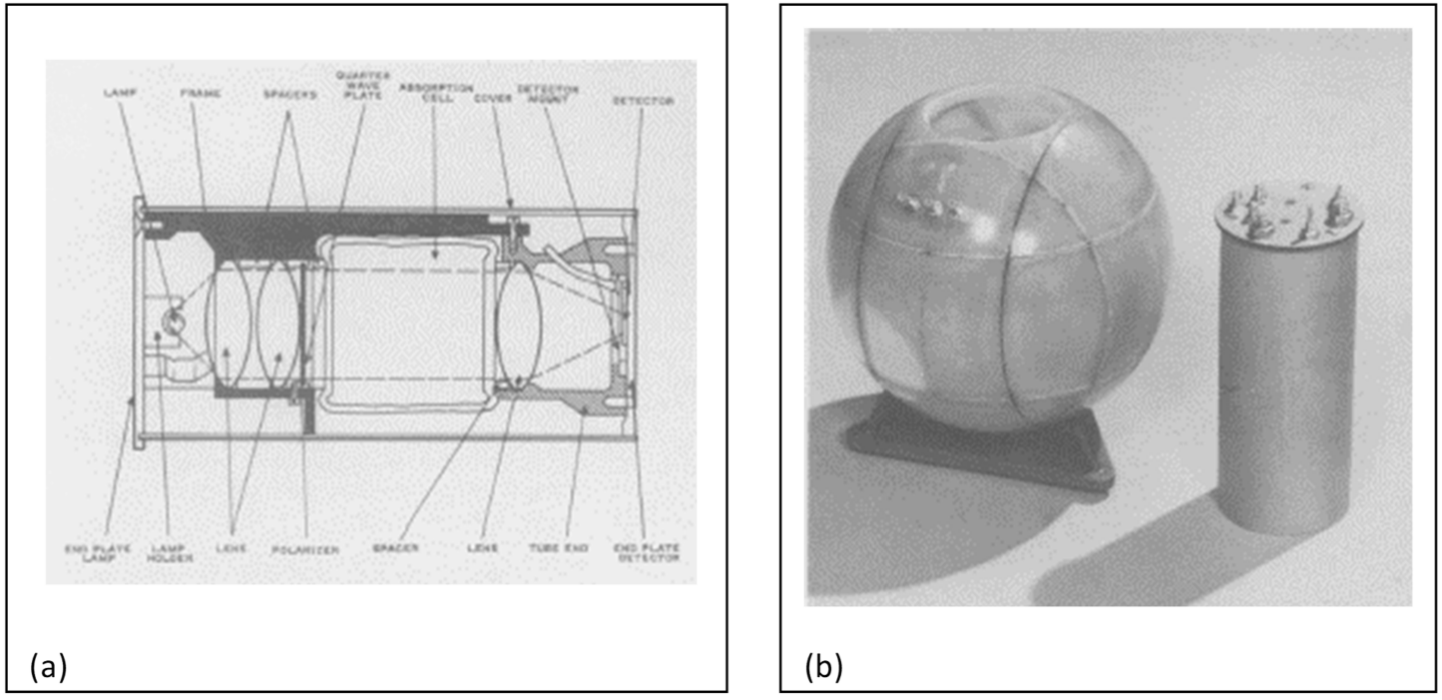
Mariner IV Vector Magnetometer Engineering Development Unit. (a) Schematic of the magnetometer head, (b) Optical pumping unit and magnetic sweep coil [1]

Quantum sensing is not new to NASA. In 1964, NASA’s Mariner IV was the first U.S. mission to fly a quantum sensor: an optically pumped vector magnetometer. Collimated, circularly polarized light from a helium spectral lamp excited quantum states of atoms in a helium cell within a magnetometer head as shown in Fig. 1 (a). This head was placed within three sets of Helmholtz coils (the optical pumping unit shown in Fig. 1 (b)) to provide a highly uniform magnetic field within the sphere. An external, ambient magnetic field disturbed the metastably pumped atoms within the cell and increased the absorption of the pumping beam through the cell, resulting in a decreased detected intensity at the end plate, whose magnitude varied with the angle of the magnetic field with respect to the optical axis. Because this magnetometer was designed to detect very low levels of ambient magnetic fields, a nulling scheme was used to measure the small intensity variations. The currents applied to the Helmholtz coils were modulated sinusoidally; in the absence of an ambient magnetic field the detected signal intensity would have the same frequency as the applied modulation. An ambient field introduced a signal deviation proportional to the ambient magnetic field strength and was measured by the amplitude of the current needed to null the deviation [1]. The first results from this sensor were provided in a 1965 press release [2].

**Figure 2:**
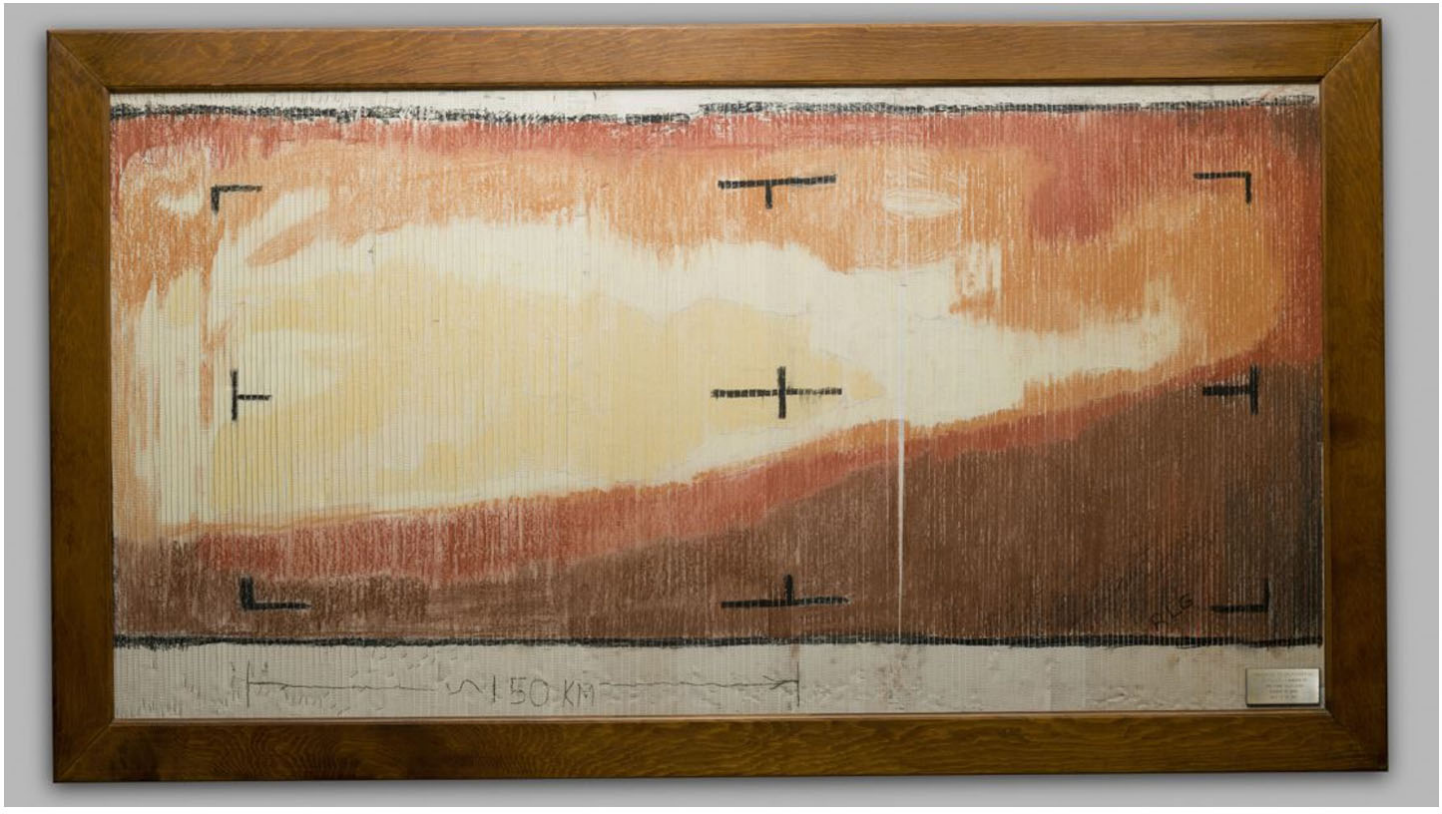
Mariner IV data reconstructed by NASA Jet Propulsion Lab (JPL) engineers in 1964 showing the first image of the Mars surface. Image Credit: NASA JPL

Variations of this optically pumped vector magnetometer were also flown on Mariner V in 1967 and Pioneer 10 in 1972 [3, 4]. Of note, Mariner IV was the first spacecraft to explore Mars and gave humanity its first close-up glimpse of another planet (Fig. 2).

### Atom interferometry

**Figure 3:**
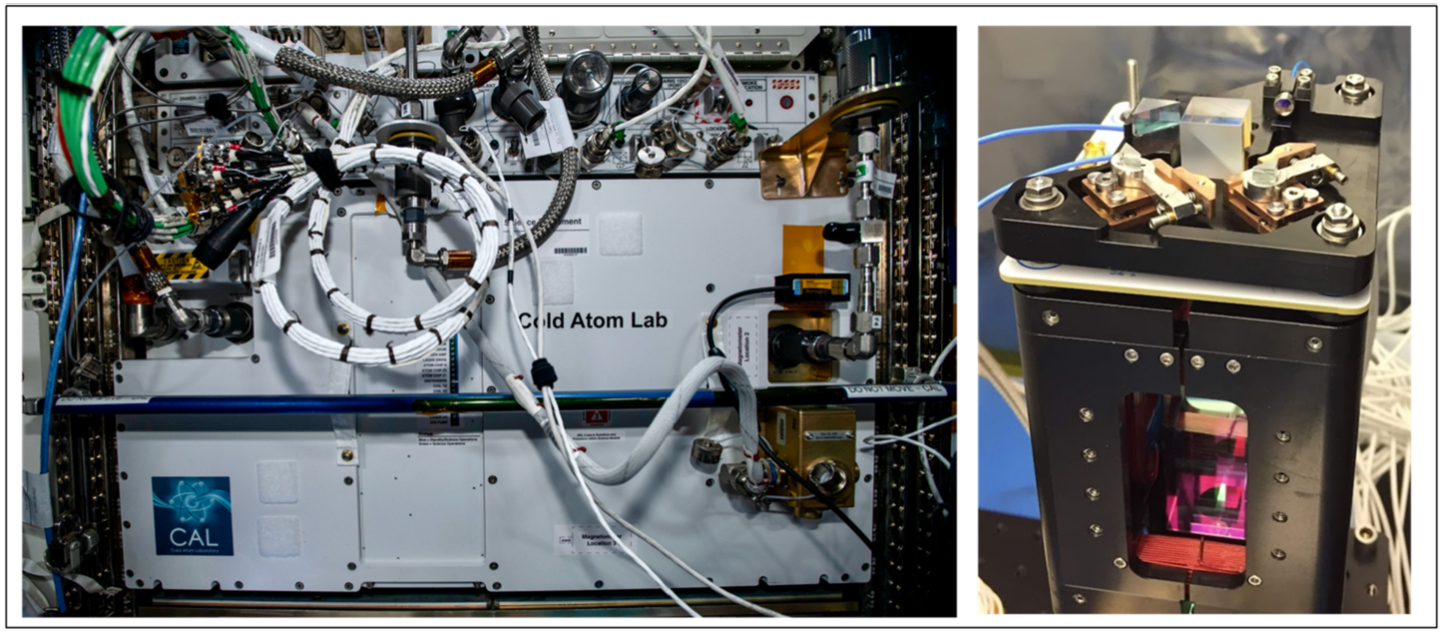
Left: Cold Atom Lab facility on the International Space Station. Right: Physics package at the heart of CAL with the atom interferometer optical platform mount on top of an ultra-high vacuum science cell. Image credit: NASA JPL

The Cold Atom Lab (CAL) on the International Space Station (ISS) is a multi-user facility for fundamental physics studies using ultracold atoms. The lab has been operational since 2018 and has demonstrated that the microgravity environment is very effective in reducing decoherence times for quantum experiments. Such experiments involve creating Bose-Einstein condensates with freefall times exceeding a second [5], persistent ultracold gases of atomic bubbles [6], and stable dual-species matter-wave interferometry [7]. While experiments in CAL typically study quantum matter at extremely low temperatures and in extended freefall to provide fundamental understanding of modern physics concepts, a Rubidium-87 (Rb-87) matter-wave interferometer was recently used as a quantum sensor to measure vibrations on the space station [8]. The interferometer uses a Mach-Zehnder design to cause atoms in trapped Rb-87 Bose-Einstein condensates to interfere by applying three retro-reflecting Bragg laser pulses to split the atoms into a superposition of two states, recombine the states, and finally read out the resulting interfering pattern in free fall. The right-hand panel of Fig. 3 shows the ultra-high vacuum science cell within CAL with the optomechanical platform above, used to direct the Bragg laser to the atoms. The science cell is 2.3 cm square by 6 cm high, fitting within the 0.4 m^3^, 230 kg CAL payload, which is shown installed in the ISS on the left-hand panel. Results from this work are leading to insights that will inform NASA’s use of atom interferometry for future remote sensing missions.

### Single photon detection

#### Superconducting nanowire single-photon detectors

**Figure 4:**
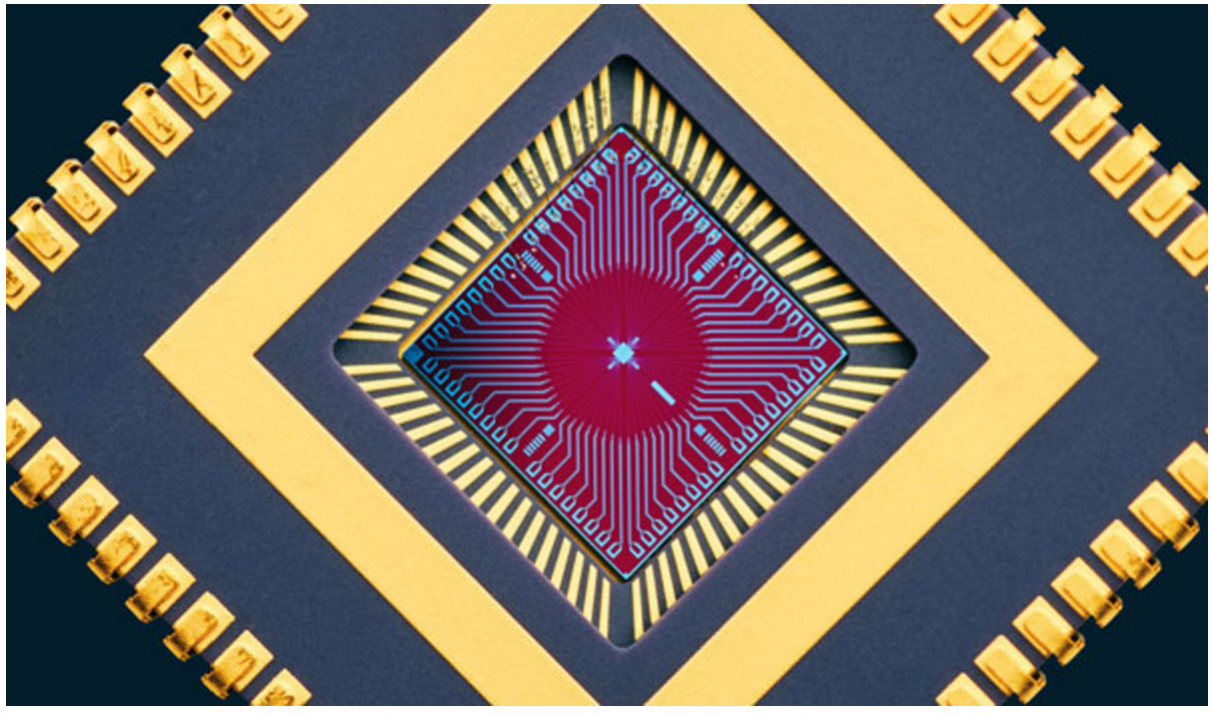
A 64-pixel SNSPD array capable of counting over 1 billion photons per second with time resolution below 100 ps. The array is mounted in a chip carrier and can be efficiently coupled to a 5-meter telescope. Image Credit: NASA JPL

NASA has been developing superconducting nanowire single-photon detectors (SNSPDs) to detect very faint astronomical signals such as those from distant stars and exoplanets. Our first use of these ultra-sensitive detectors has been as a key element in the ground receiver for humanity’s first deep space optical communications system, known as DSOC. DSOC is a technology demonstration on NASA’s Psyche mission to explore the eponymous metal asteroid in the main asteroid belt, and as of this writing DSOC is transmitting optical communications signals to Earth from beyond Mars (about 2.7 AU) at 8.3 Mbps. These faint signals are detected by the Hale telescope at the Palomar Observatory by an SNSPD array designed to cover a large area with a high-count rate. Figure 4 shows the active area of 64 SNSPD nanowires divided into four quadrants. The design includes an optimized fill factor to maximize optical absorption while minimizing the kinetic inductance and crosstalk between pixels. As part of the larger DSOC ground laser receiver system, the detector assembly enables links at data rates up to 267 Mbps. Using direct readout of each pixel limits the array size to about 100 pixels [9]. A new, time-domain multiplexing readout process has been developed and work is underway on a 400,000-pixel array for use as an ultra-sensitive detector on space telescopes [10].

NASA is working with the commercial sector as well on SNSPD development for use in a low-cost optical ground receiver for deep space communications. The receiver uses commercial SNSPDs configured with optical fiber-coupled inputs, operates at 1.55 microns, and the cryogenically cooled system is packaged in a connectorized box to be rack mountable. The system’s scalable design has two fundamental architectures as building blocks, one with a photonic lantern coupled to seven single pixel SNSPDs and one with a 16-element SNSPD array coupled to an individual few-mode optical fiber. The fiber and SNSPD components of the system have been field tested. A copy of this receiver has been adopted by the Australian National University and is planned to be used with NASA’s Optical Artemis-2 Orion and DSOC technology demonstrations [11].

#### Microwave kinetic inductance detectors

Microwave Kinetic Inductance Detectors (MKIDs) measure the surface impedance of superconducting materials as entangled electron bonds (Cooper pairs) are broken by the energy of incoming photons. The superconducting material is then used as an inductor in a resonant circuit, with the energy of the photon indicated by a phase shift in the circuit. Each inductor-capacitor circuit forms a pixel, and up to 2000 pixels can be read with a single cable and amplifier by using frequency domain multiplexing, yielding both single photon counting and energy resolution [12, 13].

**Figure 5:**
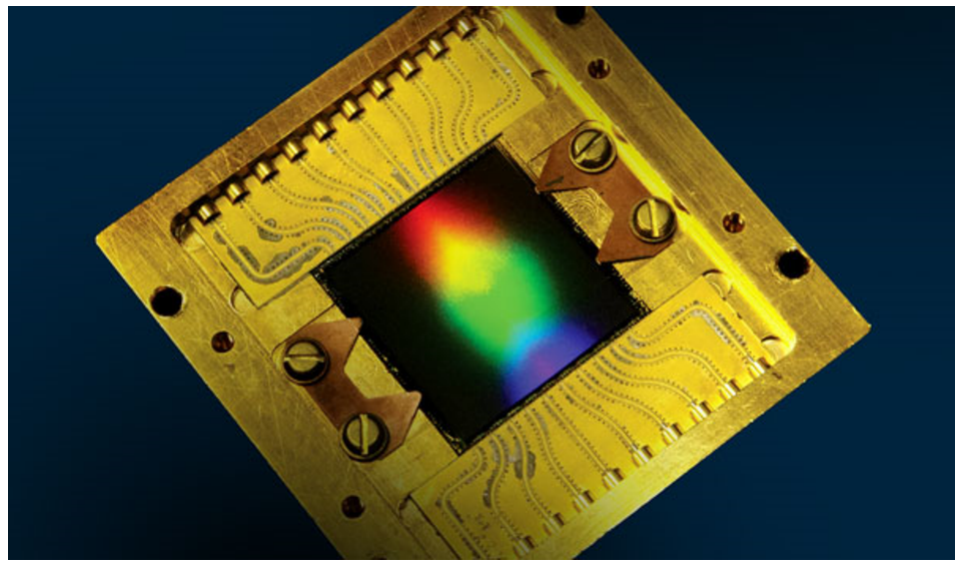
MKID array: 140 X 146 pixels, 150 um pitch, 22 X 22 mm. Image credit: NASA JPL

**Figure 6:**
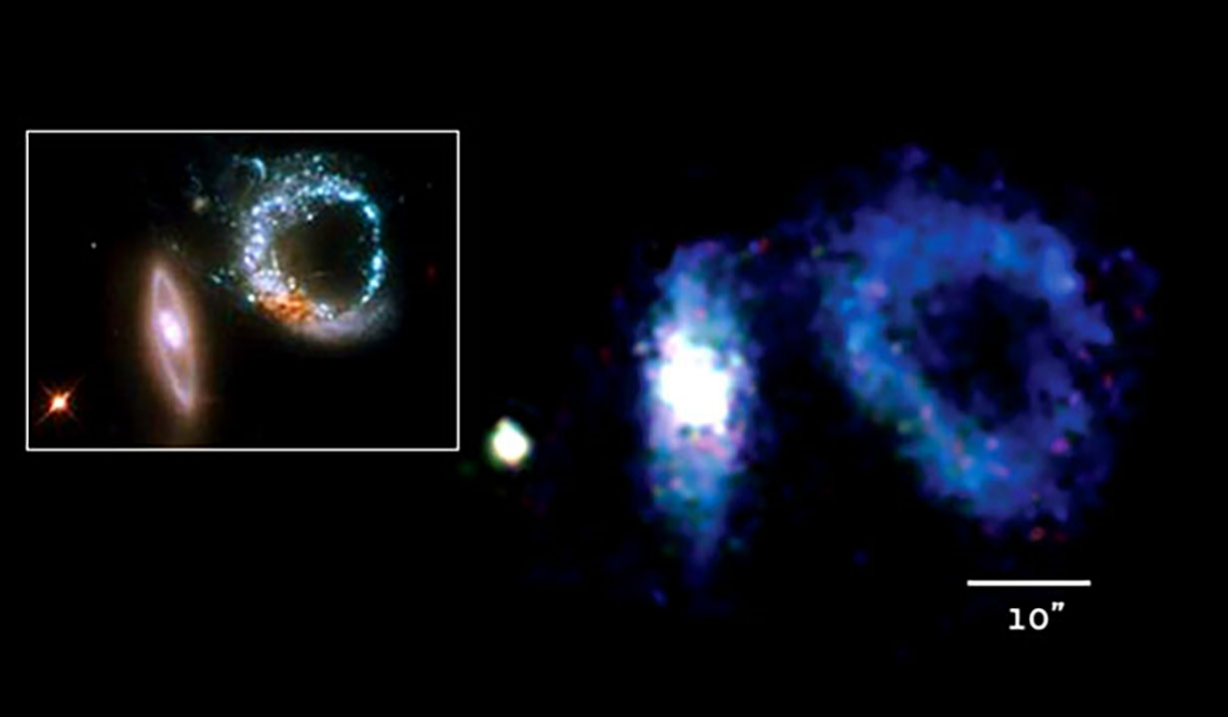
Mosaic of interacting galaxies was taken with ARCONS. The inset shows the same field of view from the Hubble Space Telescope using multiple exposures through color filters. Image credit: Hubble Space Telescope (left), ARCONS (right), overall image JPL

The primary advantages of MKIDs over conventional detectors are the fast readout time—the equivalent of several thousand frames per second—and the absence of read noise and dark current. Ultraviolet-optical-near infrared MKID detector arrays are now being used in the Hale telescope at Palomar Observatory and the Subaru telescope at Mauna Kea Observatory [14, 15]. The former uses a 2000-pixel array and the latter a 10,000-pixel array. A newer version, with 20,000 pixels, is shown in Fig. 5 and will be used for future exoplanet observations at the Subaru telescope. These detectors act as both science cameras and focal-plane wavefront sensors, enabling higher contrast ratios between a bright star and a nearby faint exoplanet. Figure 6 shows an image from the MKID sensor on the Hale telescope (Array Camera for Optical to Near-IR Spectrophotometry [ARCONS]) with each pixel containing an instantaneous wavelength spectrum [16]. MKID technology is included in the Probe Far-Infrared Mission for Astrophysics (PRIMA) mission which is one of the concepts NASA is funding for further study. Should the mission be confirmed, it would launch in 2032 to study the far-infrared universe with a 1.8 m telescope.

### Transition-edge sensors

Transition-edge sensors (TES) make use of the strongly temperature-dependent superconducting phase transition. This transition enables extremely precise measurements of small heat inputs such as those provided by individual photons. These sensors can provide 100 times better energy resolution compared to traditional detectors and can resolve key emission lines that are not detectable using more traditional approaches.

**Figure 7:**
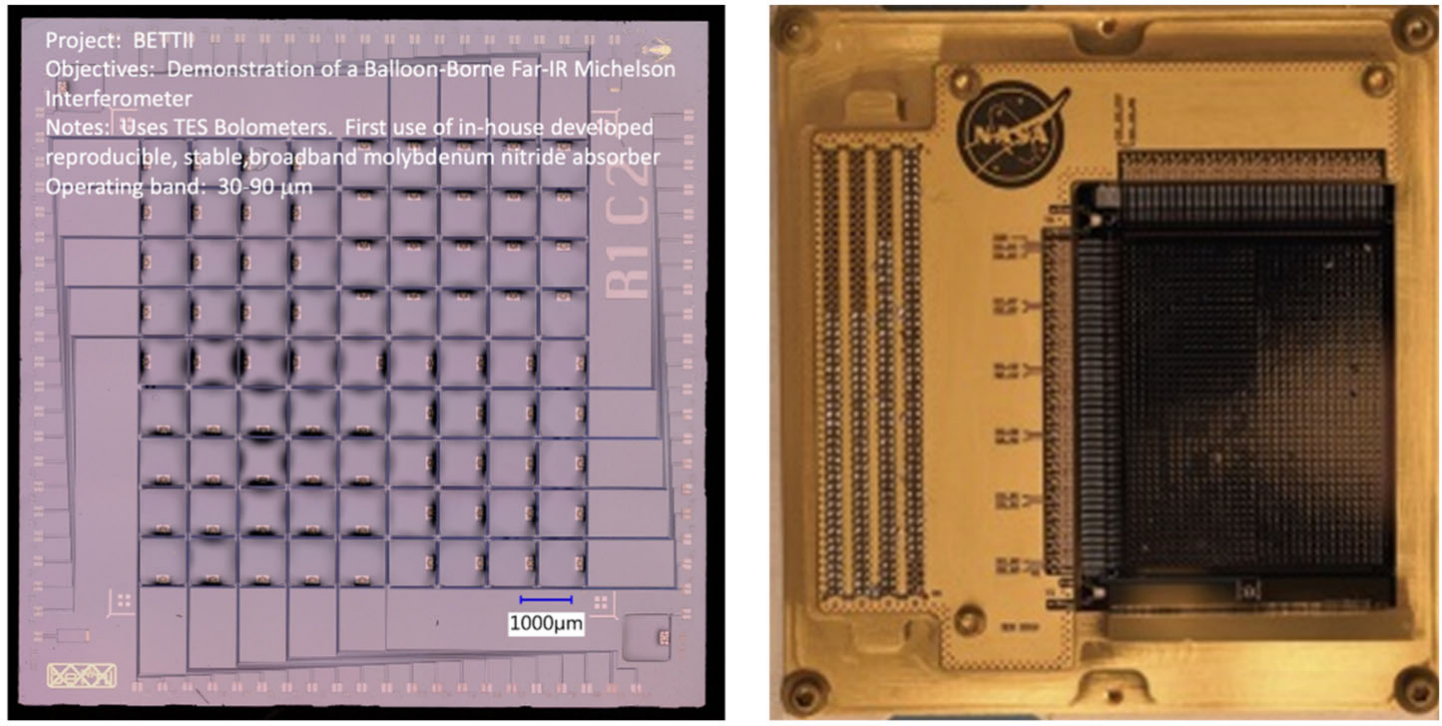
Left: Molybdenum/Gold Transition Edge Sensor built for BETTII balloon-borne interferometer experiment [20]. Right: Packaged detector wafer including a 32x40 BUG TES array for the PIPER mission [21]

**Figure 8:**
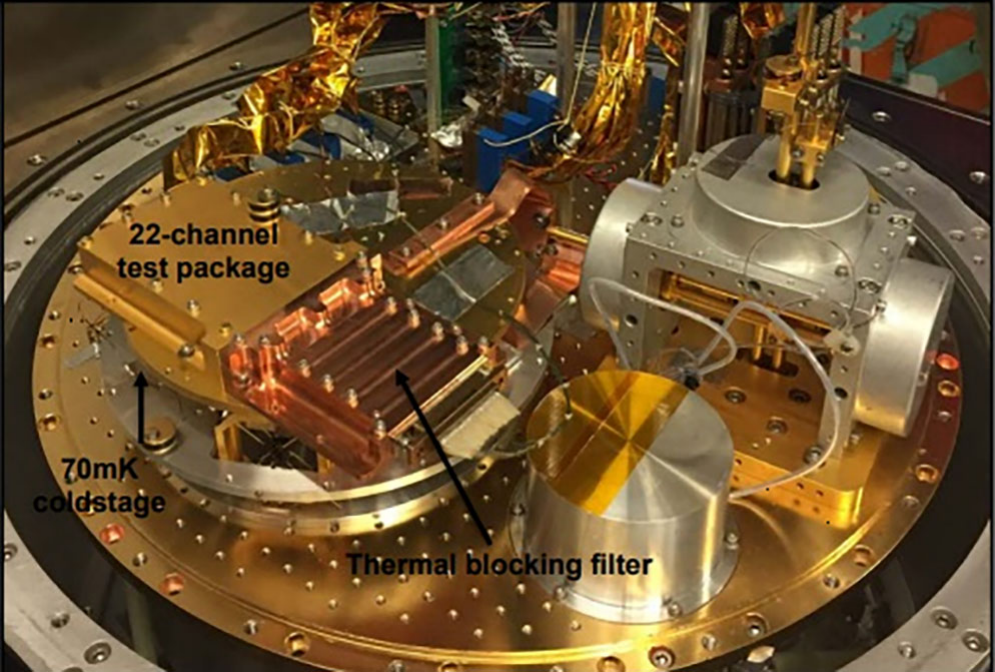
HIRMES test package mounted for characterization [22]

TES sensors have been used at least since 2007 on ground-based telescopes [17]. NASA has developed TES sensors for several suborbital flights, including two balloon flights and one airborne sensor. A TES bolometer was flown on the Balloon Experimental Twin Telescope for Infrared Interferometry (BETTII) as a technology demonstration for future interferometric missions to study galactic clustered star formation. As shown in Fig. 7, BETTII’s detector was based on a reproduceable, stable broadband molybdenum nitride absorber operating in the 30 – 90 micron band. Another TES sensor developed for a balloon flight is also shown in Fig. 7: the low-noise backshort-under-grid (BUG) kilopixel sensor array. This 32x40 element detector is included in each of the twin telescopes on the Primordial Inflation Polarization Explorer (PIPER) balloon experiment to map the polarization of the cosmic microwave background [18]. Finally, the Stratospheric Observatory for Infrared Astronomy (SOFIA) carried several instruments during its twelve years of operation. Developed and tested for use on SOFIA, the High Resolution Mid-Infrared Spectrometer (HIRMES) used a TES bolometer as the detector element to detect neutral atomic oxygen, water, and heavy hydrogen between 25 and 122 microns to study the composition and kinematics of protoplanetary disks and their evolution into young solar systems. The HIRMES focal plane is comprised of two 16x32 low-resolution and one 6x16 pixel high-resolution detector arrays with a superconducting quantum interference device (SQUID) multiplexing readout [19]; its assembly ready for testing is shown in Fig. 8. HIRMES completed integration and testing but was not flown before SOFIA’s cancellation in 2022.

### Single X-ray detection

**Figure 9:**
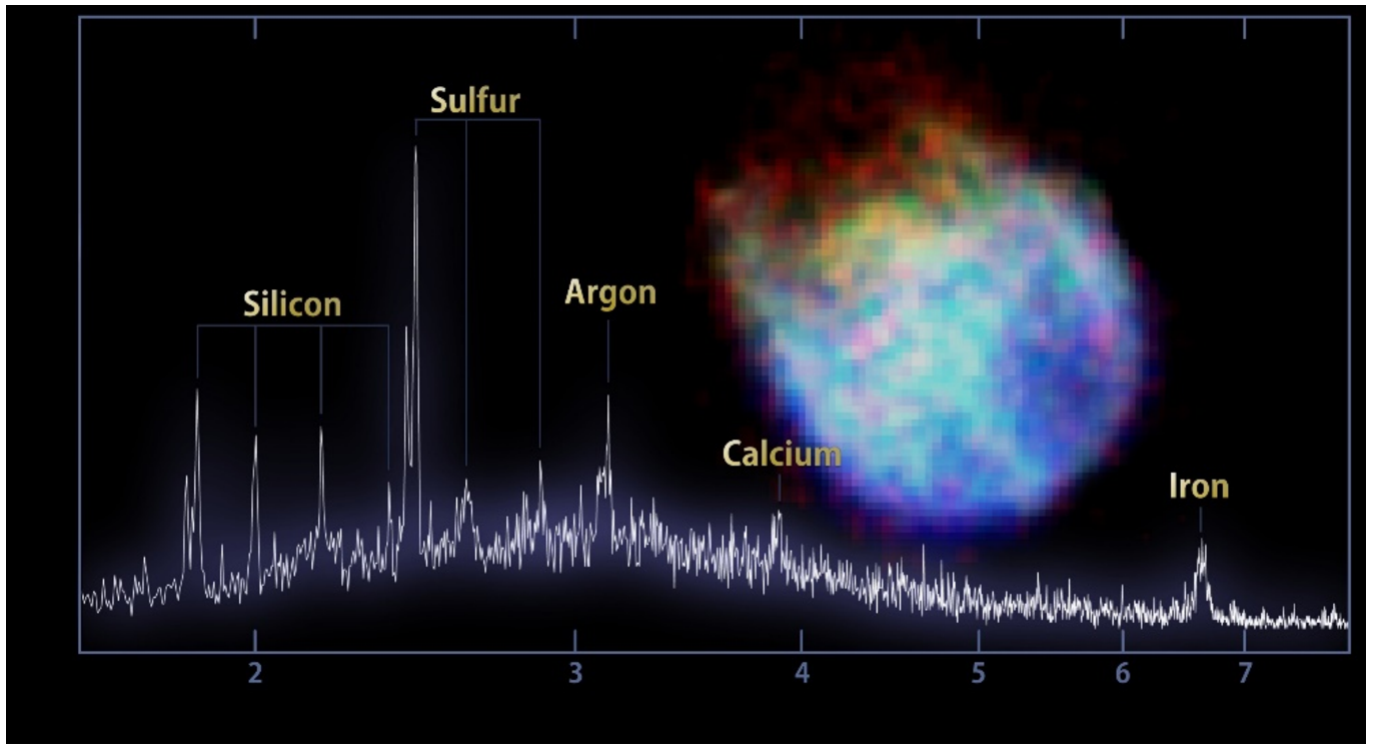
Supernova Remnant N132D spectral data from the Resolve spectrometer on XRISM. The horizontal axis shows X-ray energy in keV; the vertical axis shows relative X-ray brightness. The image at right was captured by XRISM’s Xtend instrument. Image credit: JAXA/NASA/XRISM

NASA has also developed calorimeters to measure X-ray radiation. The path to application for this technology took decades, although the delay was not caused by the technology but rather the scarcity of flight opportunities for X-ray missions. In 1976 NASA selected the Advanced X-ray Astrophysics Facility as a new Great Observatory mission to study the structure and evolution of the universe. As the mission matured it was renamed the Chandra X-ray Observatory and launched in 1999. However, budget pressures required a reduction in the number of new technologies that could be flown on Chandra and the quantum microcalorimeter that was originally included on the mission was omitted [23, 24]. The quantum microcalorimeter was redesigned and selected for inclusion on the Japanese Astro-E X-ray observatory [25] that launched in 2000 but failed to reach orbit. It was also included on the Astro-E2 Suzaku mission that launched in 2005, but spacecraft interface failures prevented instrument use. A third attempt in the series, Astro-H Hitomi, yielded a week’s work of science operations [26] before that spacecraft was also lost. Success came with the Japanese XRISM mission. The quantum microcalorimeter developed by NASA’s Goddard Space Flight Center (GSFC) in the 1980 s was launched in 2023 as the detector for the Resolve X-ray spectrometer onboard XRISM [27]. The data from Resolve depicted in Fig. 9 shows the most detailed X-ray spectrum ever obtained from a supernova remnant in the Large Magellanic Cloud.

**Figure 10:**
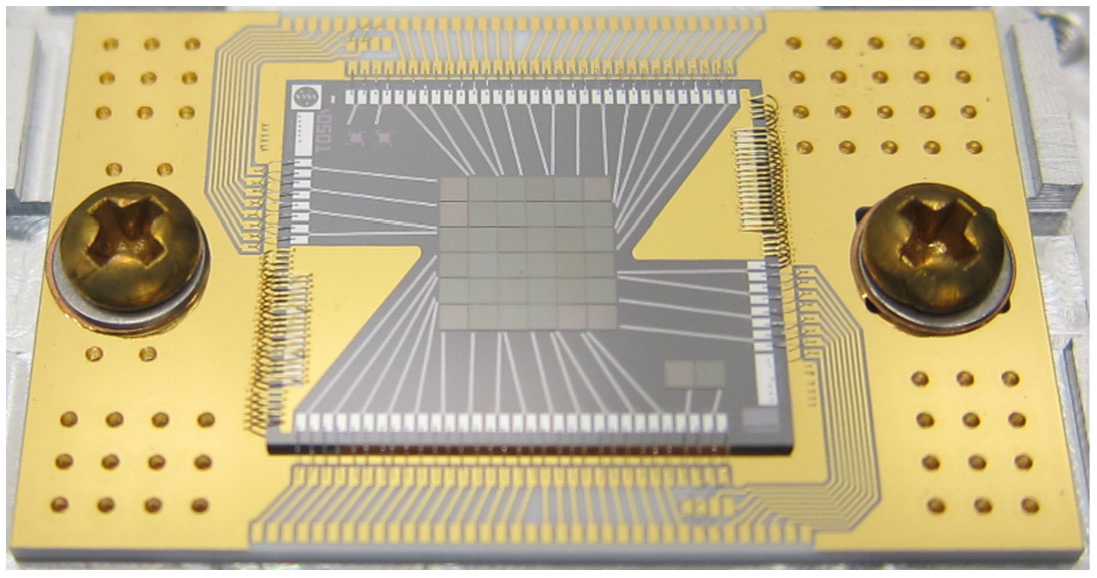
Photo of the XRISM/Resolve quantum-calorimeter array in its storage container prior to integration into the instrument. The 6x6 array, 5 mm on a side, consists of independent detectors – each one a thermally isolated silicon thermistor with a HgTe absorber. Image credit: NASA GSFC

The quantum calorimeter on Resolve is a 6x6 detector array based on silicon-thermistor thermometers and mercury telluride (HgTe) X-ray absorbers [28] as shown in Fig. 10. It makes precise measurements of energy quanta by measuring the temperature change that occurs when a quantum of energy is deposited in the HgTe absorbers that were designed by EPIR Technologies to provide a very low heat capacity. The thermistor is designed to significantly reduce low-frequency noise, include reproducible connections between absorbers and thermistors, and enable compact thermal isolation to make square arrays feasible. To reduce thermodynamic noise and the heat capacity of the sensor, operation at temperatures less than 0.1 K is required.

### Atomic clocks

Atomic clocks have been flown on US spacecraft since 1974 when the Naval Research Laboratory launched Navigation Technology Satellite 1 (NTS-1, aka Timation 3) with a rubidium-vapor atomic clock to demonstrate advanced navigation technology. Not only did this spacecraft demonstrate precision timekeeping for navigation it also extended the ground-breaking demonstration of Einstein’s general and special theories of relativity from aircraft to spacecraft by showing differences in clock rates based on speed and altitude [29]. NTS-2, with a cesium-based atomic clock, became the prototype for today’s Global Positioning System (GPS) which continues to use atomic clocks to provide accurate surface position data for a multitude of commercial and government uses [30]. Earth science researchers use GPS as a remote sensing tool to support atmospheric and geodetic sciences, including monitoring sea levels, measuring the Earth’s rotation, and quantifying regional deformations caused by tectonic shifts [31].

Ultra-stable oscillators have been included on a variety of NASA missions as stand-alone instruments to enable radio science. Examples include Doppler wind experiments on the Galileo probe launched in 1989 to study Jupiter and Cassini’s Huygens probe launched in 1997 to study Titan. A more complete listing is found in Asmar’s 2022 paper [32].

**Figure 11:**
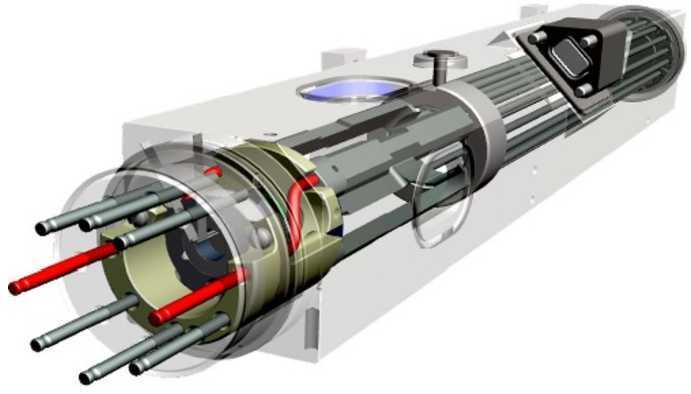
Deep Space Atomic Clock. Image credit: NASA JPL

The GPS network of satellites orbit the Earth at about 20,000 km making them unsuitable for navigational use in cislunar space and beyond. NASA developed the Deep Space Atomic Clock (DSAC), shown in Fig. 11, to demonstrate a highly precise clock that could enable independent navigation for deep space missions as well as highly accurate radio science. DSAC used electromagnetic fields to confine mercury ions. It was launched in 2019 and demonstrated an order of magnitude improvement in space clock performance with long-term fractional frequency stability of 3 × 10^−15^ at 23 days, and an estimated drift of 3.0 × 10^−16^ per day [33].

In April 2025 the European Space Agency sent the Atomic Clock Ensemble in Space (ACES) to the International Space Station. ACES will compare its on-board cesium and hydrogen-maser clocks to highly accurate clocks on Earth and is expected to achieve a fractional stability and accuracy of 1 × 10^−16^. This will enable it to conduct precision tests of Einstein’s theory of general relativity, search for time variations of fundamental constants of physics, and test extensions of the standard model of particle physics [34].

## Current investments

### Astrophysics

Astrophysical sciences can significantly benefit from the advantages and diverse properties of quantum sensing because distant sources, such as those found in the primordial universe, produce signals of interest that are in an ‘information-starved’ regime (i.e., require single photon detection). For these observations of the distant universe, the detection levels are already in the quantum regime of single photons arriving at the telescope focal plane, which make them suitable for acquisition by superconducting quantum devices.

To achieve the next level of detection in astrophysics, sensing technology demands the exploitation of quantum effects to obtain the highest sensitivity observations allowed by nature. The search for life on distant planets, for example, will demand the use of superconducting detectors because of their superior noise performance compared to semiconductor detectors such as charge-coupled devices and complementary metal-oxide semiconductors.

Consequently, most NASA astrophysics investments to date have focused on quantum technologies related to single-photon detectors, ultrasensitive bolometers, and quantum calorimeters. These technologies use quantum effects such as superconductivity, quantum interference, quantum capacitance, quantum tunneling and quasi-particle trapping to achieve high sensitivity and optimal performance.

**Figure 12:**
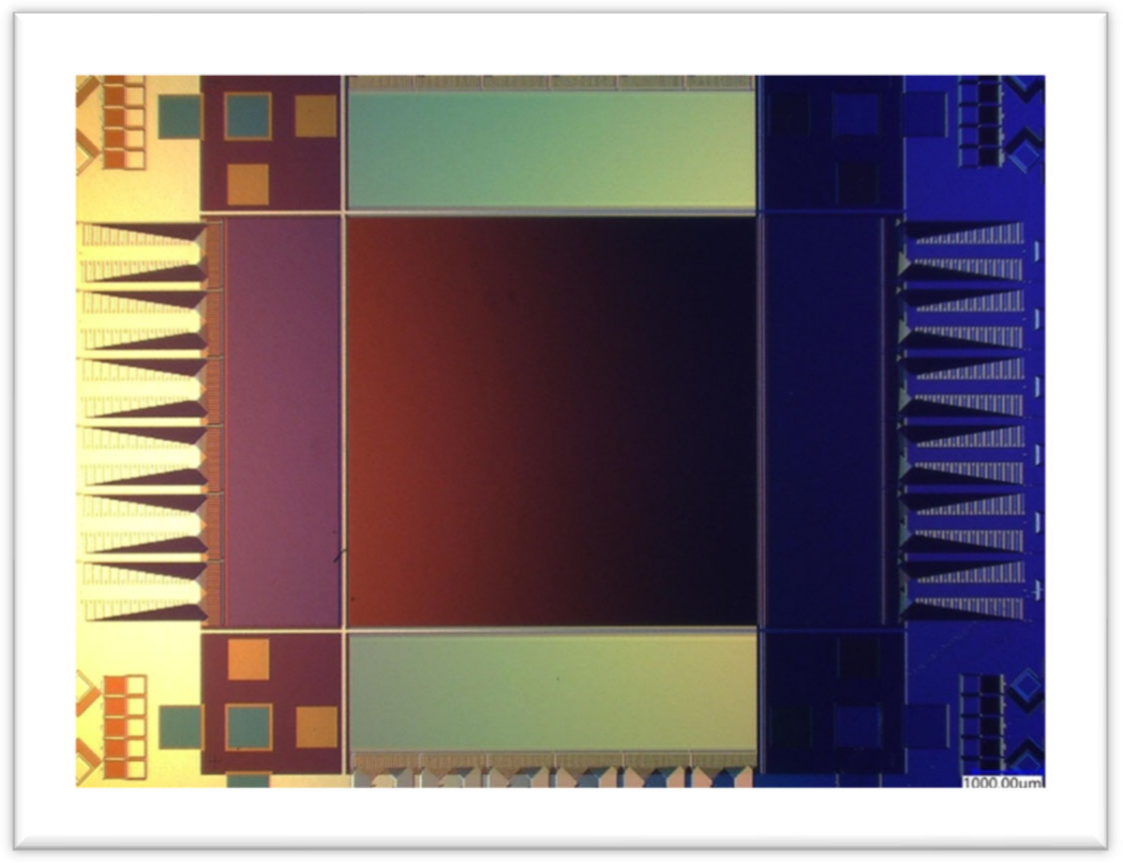
The 400,000 pixel superconducting camera based on superconducting-nanowire single photon detectors. Credit: Adam McCaughan/NIST

As described in Sect. 2, *SNSPDs, MKIDs, TESs, and quantum microcalorimeters* are already making substantial improvements to astrophysics measurements and NASA is investing in performance improvements for each. SNSPD technology has been advanced from the 64-pixel detector used to receive signals from DSOC to the development of a camera with 400,000 pixels in collaboration with researchers at the National Institute of Standards and Technology (NIST), as shown in Fig. 12 [35]. NASA is also funding an effort to develop higher performance SNSPDs based on transparent superconducting thin films. These new materials offer the promise of improved efficiency and signal-to-noise ratio, and reduced detector deadtime and intrinsic timing jitter [36].

Through the use of TES thermometers, the quantum microcalorimeter technology developed for Resolve as described in Sect. 2 has been expanded to 2000 pixels for the European Space Agency (ESA) Advanced Telescope for High Energy Astrophysics (ATHENA) mission to study high energy astrophysical processes, and a collaboration between GSFC, the Massachusetts Institute of Technology Lincoln Laboratories, and the National Institute of Standards and Technology is underway to create a 100,000-pixel version using both TES and paramagnetic thermometers [37].

MKID technology is being advanced through several efforts. The floor level for the noise equivalent power for MKIDs was reduced to around to 10^−20^ WHz^−1/2^, a performance sufficient for use on the proposed PRIMA probe mission [38]. The spectral resolution of MKID detectors is being improved by reducing amplifier noise and altering the acoustic properties of the superconductor-substrate interface [39]. Work is underway to commercialize this technology via small businesses: MKID sensors are being integrated into on-chip spectrometers [40]and a scalable data acquisition system for MKID detectors is being developed [41].

Many of these technologies are critical for future X-ray and far-infrared (IR) missions, including upcoming mid-size strategic missions and larger flagship missions such as the Habitable Worlds Observatory (HWO). However, energy resolving MKIDs do not yet meet plausible energy resolution requirements, and TESs with energy resolving capabilities are not available in large format arrays. Likewise, SNSPDs are not able to resolve energy, and since all these detectors require cryogenics devices, the demand for energy resolving capabilities for space applications could make MKIDs and TESs the preferred devices for space astrophysics applications; therefore, NASA will continue its investments to advance these technologies.

### Earth science

NASA’s Earth Science Division is investigating several quantum-related activities to significantly improve our current measurement capability and/or enable new measurements to understand the Earth as a system.

*Quantum Gravity Gradiometer Pathfinder*: Gravity measurements are critical to drought monitoring, water management, determination of flood potential, and many other applications that have important implications for agriculture, industry, security, and everyday life.

Measuring changes in how mass is distributed on and beneath Earth’s surface, particularly in the form of water and ice, are fundamental indicators of the large-scale dynamics of the planet. By measuring tiny gravitational changes over timescales of days to months to years, scientists can observe the movement of ice sheets and glaciers, monitor the rise and fall of underground aquifers, detect changes in the levels and currents of the oceans, gauge the amount of water in large lakes and rivers, and even discern underground features of interest.

**Figure 13:**
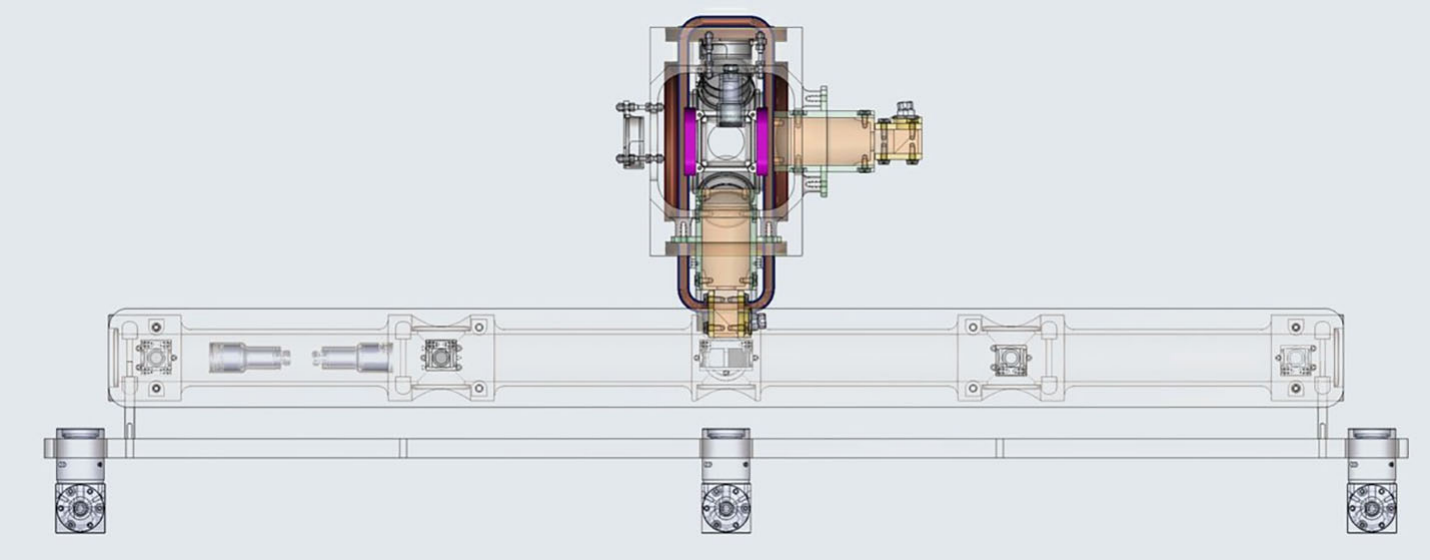
Sketch of a notional concept for a single source design of the pathfinder payload based on a Rubidium source. (Credit: NASA JPL)

Early work funded by NASA showed the feasibility of using two stacked atom interferometers for absolute-gravity gradiometry and identified the expected benefit of long interrogation times afforded by a microgravity environment [42]. NASA is now developing a Quantum Gravity Gradiometer (QGG) pathfinder instrument, with the aim to deliver an instrument for on-orbit testing no earlier than 2030 [43]. A notional schematic of an instrument reference design is shown in Fig. 13.

Using cold atom interferometry, the QGG has the potential to collect more precise measurements of Earth’s gravitational field than existing methods—such as the satellite-to-satellite tracking utilized by NASA’s Gravity Recovery and Climate Experiment (GRACE) and GRACE-Follow On missions [44]—and could do so from a single satellite.

One of the largest components of an atom interferometer is the ultra-high vacuum system needed to interrogate atoms over extended periods or with large separations. NASA is funding Q-Peak, Inc. to develop an ultra-high vacuum chamber using an aluminum alloy to significantly reduce the weight and passive pumping based on non-evaporable getter pumps to reduce the power consumption [45].

*Rydberg Sensing instruments*: Rydberg atomic sensors are highly sensitive broad-spectrum quantum detectors that can be dynamically tuned to sense micrometer-to-millimeter waves with no requirement for radio frequency (RF) band-specific electronics. Rydberg atomic sensors can use existing transmitted signals such as those from navigation and communication satellites to enable remote sensing over a broad spectrum. NASA is currently investing in two Rydberg sensing instrument development efforts:

*Quantum Rydberg Radar*: NASA is investing in quantum Rydberg radar technologies with the potential to enable high-sensitivity, dynamic, and rapidly tunable radar remote sensing across the radio window without the need for specialized science antennas, or RF front-end electronics. The rapid tunability and the ability to leverage existing satellite signals instead of an on-board transmitter (known as signals of opportunity, or SoOp) could enable a lower cost architecture for making new measurements in science domains like land surface hydrology.

**Figure 14:**
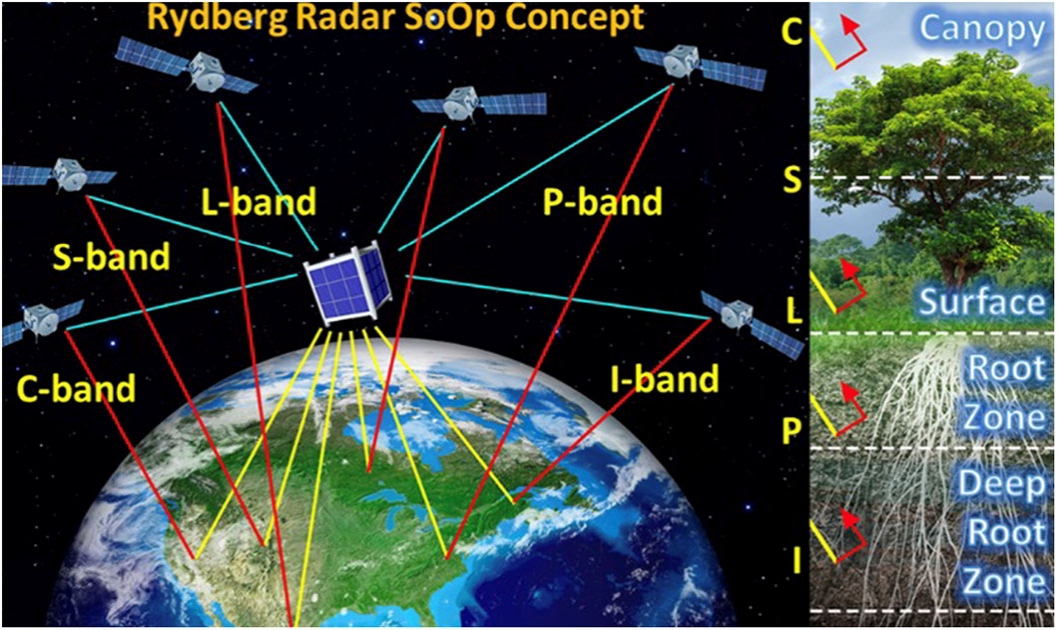
Mission concept of Rydberg Radar in view of multiple SoOp navigation and communication satellites

Figure 14 shows the concept for a broadband polarimetric radar using Rydberg sensors configured with atomic super-heterodyning to measure soil moisture using XM radio satellite signals. NASA recently completed the first demonstration of remote sensing of soil moisture, an important Earth system variable, via ground-based radar reflectometry with Rydberg atomic systems [46].

**Figure 15:**
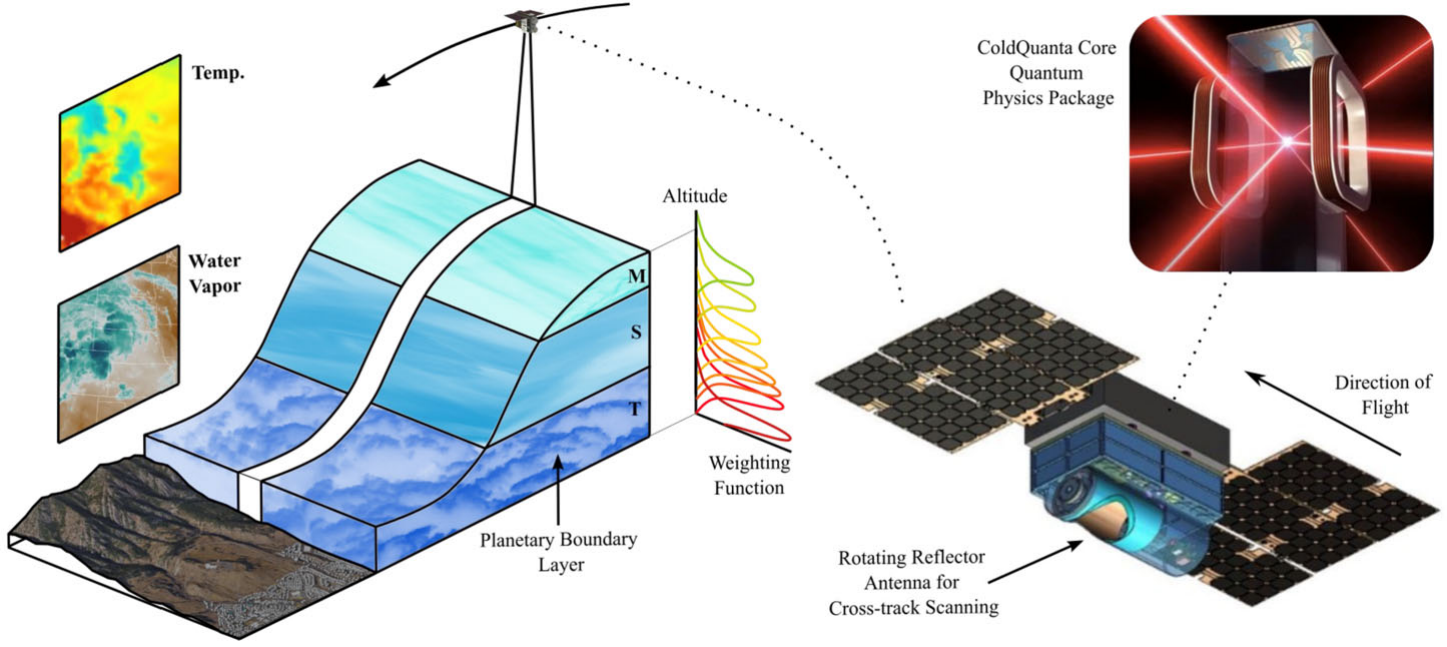
A QuARREM-based microwave radiometer flight concept. Image credit: ColdQuanta Inc. [48]

*Quantum Atomic Rydberg Radiometer for Earth Measurements (QuARREM)* is a Rydberg-based hyperspectral quantum microwave radiometer being developed for planetary boundary layer science applications. It uses a compact atomic vapor cell sensor instead of conventional radio-frequency electronics to reduce the size, weight, and power of the instrument for use on smaller spacecraft without sacrificing performance. Specific innovations include developing a system for self-calibration and expanded modeling capabilities to assess system functions [47]. Figure 15 shows a concept for an instrument employing QuARREM in a cross-track scanning mode using a rotating reflector to enable surface imaging and planetary boundary layer science.

### Planetary science

While no planetary science missions have used quantum sensors since the use of the early magnetometers described in Section 2.1, NASA is now developing several new technologies for this application. As described below, these concepts include a seismometer and accelerometer based on atom interferometry, magnetometers based on quantum color-center devices, a SQUID-based communication system, and a portable optical frequency comb for spectrometry.

**Figure 16:**
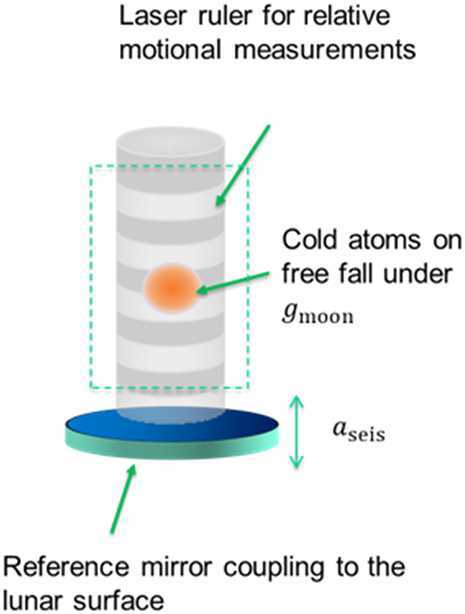
Lunar gravity and surface motion measured by a free-falling laser cooled atomic cloud in a vacuum chamber Image credit: NASA JPL

*Atomic lunar seismometer:* Measurement of the Moon’s seismic and gravitational properties would enable the study of the lunar interior, core, and structure. A low measurement frequency (<10 mHz) cold atom interferometer is being developed to probe the interior and structure of the Moon by measuring seismic waves, long-period global normal modes, and gravity. NASA is developing a lunar atomic seismometer instrument that employs atomic test masses as the inertial reference frame and atom interferometry to enable displacement measurements. The atomic lunar seismometer shown in Fig. 16 is designed to be a landed instrument, operating under the Moon’s gravity with atomic test masses (rubidium) moving relative to the instrument hardware to enable ultra-low frequency measurements—a scenario that requires a longer duration mission. The instrument’s mirror will be rigidly coupled to Moon’s surface to detect seismic activity. The advantages of this technology over the state of the art are that there are minimal to no thermal and mechanical drifts, in-situ calibration is not necessary, and improved performance is expected [49].

**Figure 17:**
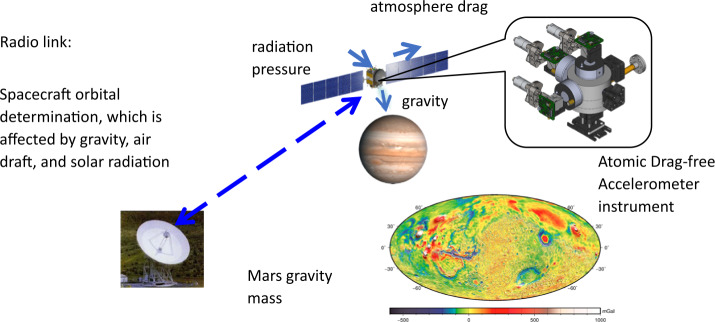
Illustration of the proposed atomic drag-free accelerometer (*ADA*) for non-gravitational drag force measurements in a typical radiometric link for spacecraft orbit determination and planetary gravity measurements. Image credit: NASA JPL

*Atomic drag-free accelerometer (ADA):* Measurement of gravitational properties for planetary bodies with heavy atmospheres or strong solar radiation can enable an understanding of the planetary density, topology, interior structure, atmospheric processes, and dynamics. A compact three-axis cold atom interferometer sensor is being developed for gravity measurements and/or non-gravitational force measurement via radiometric orbit determination from a spacecraft. The target sensitivity is less than 3x10^−8^ m/s^2^/$\sqrt{} H z$ at a frequency of less than 1 Hz. This instrument uses free-fall cesium atoms as proof masses and quantum wave interference for inertial force measurements. As shown in Fig. 17, the atomic drag-free accelerometer is designed to operate in orbit under microgravity where there is little relative motion between the atomic cloud and the spacecraft. The advantage of this technology over the state of the art is that it eliminates drag force errors, allows for lower orbits for planetary bodies with larger gravity, and should provide improved performance.

**Figure 18:**
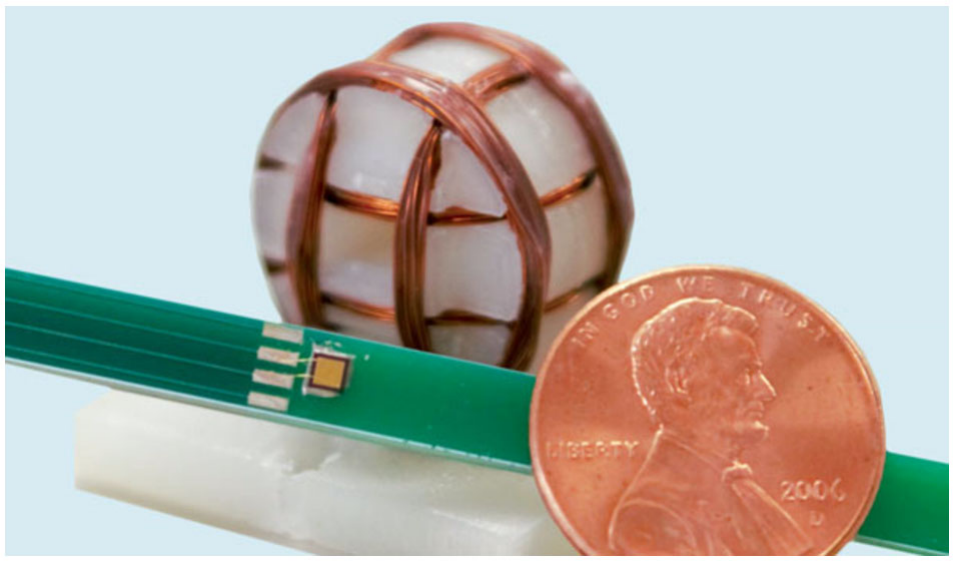
SiCMag sensor and the 3-axis, 3D printed Helmholtz coil system used to modulate and null the external field (*Image credit: NASA JPL*)

*Silicon carbide magnetometer:* Moons orbiting Jupiter and Saturn are believed to contain liquid water oceans under thick ice sheets. These ocean worlds—with plentiful heat from an interior core, liquid water, and protection from radiation—are promising locations to search for life, so understanding these oceans is a high priority for the science community. These oceans can function as a planet-sized electric circuit that can disturb and modify the measured magnetic field of a planetary body; therefore, NASA is developing an optically pumped solid-state magnetometer that can measure these very small magnetic field disturbances [50]. The silicon carbide magnetometer (SiCMag) leverages atomic-scale defects intrinsic to a silicon carbide (SiC) semiconductor to form a single color-center sensor. The novel devices use the electron-nuclear hyperfine mixing of the electron spin pairs involved in spin-dependent recombination at near-zero magnetic fields to enable the electrical readout of a spin-dependent current modified by the ambient magnetic field. The sensor consists of a SiC diode placed within three sets of orthogonal Helmholtz coils as shown in Fig. 18. The coils are used to modulate the magnetic field on each axis, each at a different frequency, and the diode generates frequency-division multiplexed vectorized magnetic field measurements. The target sensitivity is tens of *pT*/$\sqrt{} H z$, and self-calibration is achieved by monitoring the magnetic isotopes of the host and dopant atoms in the color-center sensor, which act as tiny bar magnets [51].

**Figure 19:**
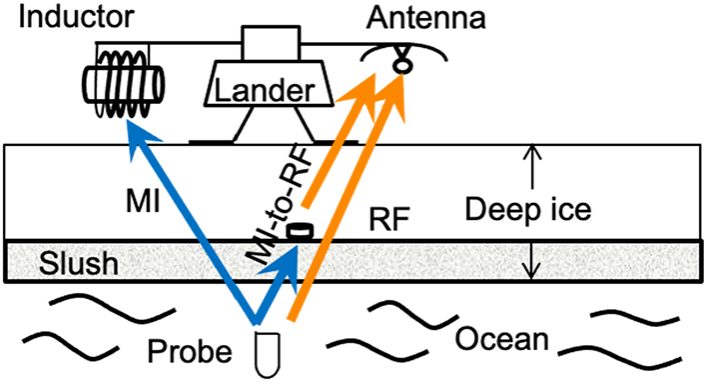
Magneto-inductive communications can penetrate through conductive media but the data rate is low, while radio frequency communications are less effective in slush but offer high data rate; thus a combination of these two technologies has the potential for most efficient communication. Image credit: NASA JPL

*Hybrid radio frequency and magneto-inductive transceiver:* Exploring ocean worlds such as Europa requires probes that can penetrate the thick top layer of ice, reach the buried ocean below, investigate the fluid environment, and transmit data back to the surface. Typical communication links rely on electrically connected wires that can be spooled from the lander on the surface of the ice to the melt probe to relay data collected while investigating the ocean below. NASA is developing a hybrid radio frequency and magneto-inductive transceiver for Europa sub-ice communications. This magneto-inductive transceiver communications system would enable information to be transmitted using a non-propagating, quasi-static magnetic field that can penetrate highly conductive media such as water and ice, as shown in Fig. 19. The magneto-inductive transceiver communications system is based on a SQUID and a chip-scale atomic magnetometer (CSAM) to enable a magneto-inductive-to-radio-frequency (RF) bridge link. Since the magnetic field strength will degrade as the distance cubed, a very sensitive receiver technology such as this is needed to pick up the weak signal after it has penetrated several kilometers of briny ice. This hybrid RF and magneto-inductive communications link will enable data transfer through ice and slush without electrically connected wires.

*Mid-infrared frequency comb spectrometer*: Many interplanetary missions are being developed to detect molecular species of interest on planetary bodies, an endeavor that corresponds to one of the key focus areas for NASA—the search for life beyond Earth. NASA is developing a mid-infrared spectrometer featuring quantum cascade laser-based optical frequency combs to detect molecular species. Space-based applications require instruments with low size, weight, and power consumption and excellent spectral performance in the 3- to 4-micron spectral region for astrobiology measurements. A semiconductor-based mid-infrared optical frequency comb was developed using an interband cascade laser; dispersion compensation techniques are being developed to extend the emission bandwidth by incorporating components such as a coated mirror or reflective grating within an external cavity and the use of adaptive optics such as a micro-electrical-mechanical mirror within the external cavity. The goal is to achieve a 200 nm spectral bandwidth with a center wavelength at 3.3 microns [52, 53].

*Microfluidic hyperpolarized enhanced nitrogen vacancy - nuclear magnetic resonance (NV-NMR):* A team at the University of Maryland at College Park is integrating multiple techniques to develop a highly sensitive instrument for non-invasively detecting single cells, metabolites, amino acids, and other macromolecular organics. The expected application would be a mission to search for signs of life on another planet. Current single cell studies rely on the amplification of nucleic acids, which is not possible for proteins and metabolites. Conventional NMR is not practical for in situ astrobiology measurements because it requires large sample volumes and high concentrations, but NV-NMR is more sensitive, enabling picoliter sample volumes and the detection of signals at nanometer spatial scales. Hyperpolarization techniques (e.g., Overhauser Dynamic Nuclear Polarization [DNP] and Para-Hydrogen-Induced Polarization [PHIP]) enhance magnetic alignment of the nuclei and slow lossy relaxation processes. Microfluidics technology allows precise sample manipulation and control of environmental factors, including single cell trapping.

*Quantum Sensor for deuterium to hydrogen (D/H) Ratio Measurement in Water:* NV-NMR is also being used to develop a sensor to study of the ratio of D/H isotopes, which would enable the determination of whether water in the outer solar system originated from comets and asteroids, or whether it was formed through processes such as solar wind implantation or outgassing from a moon or planetary interior. This information would provide insight into the early history and formation of the Moon and the inner Solar System, with significant implications for future lunar exploration and the development of sustainable space exploration.

### Heliophysics

Sensors designed to measure distant stars are also useful to gain knowledge about the Sun, so many advances being made for astrophysics will find application in heliophysics. Heliophysics is funding innovative sensors that are light enough to be mounted on solar sails that might one day travel to the outer reaches of the solar system.

**Figure 20:**
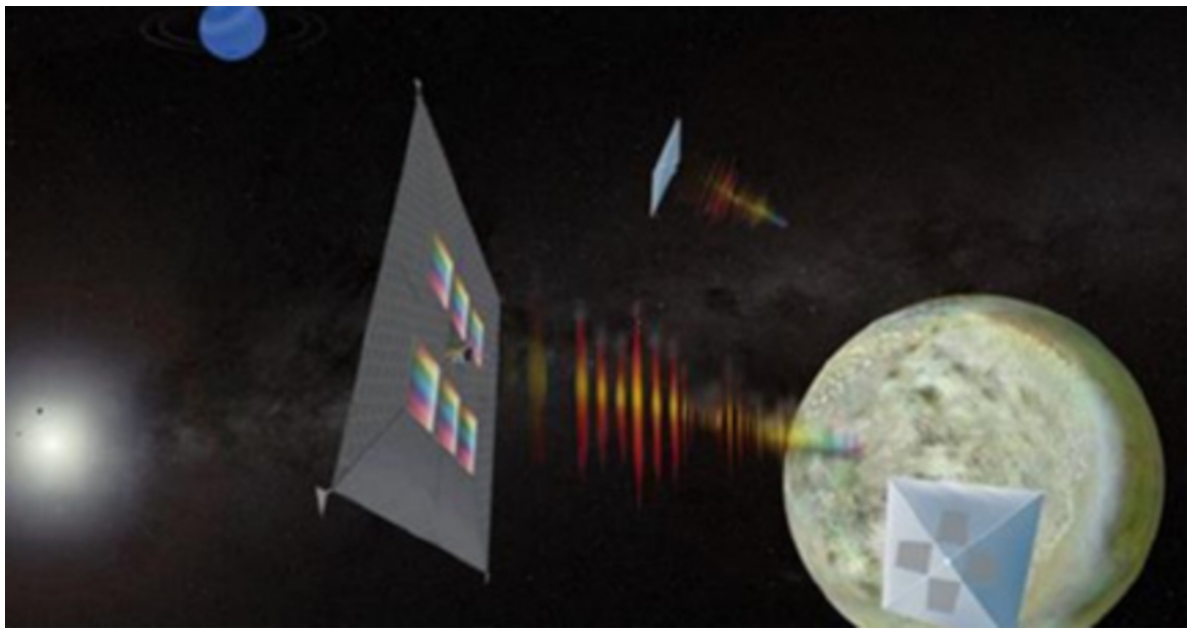
Artist’s depiction of a solar sail with quantum dot spectrometers printed directly on the sail surface to form a monolithic, lightweight structure. Image credit: NASA

*Quantum dot spectrometer:* Conventional spectroscopy requires long path lengths for gratings and prisms to achieve high spectral resolution. Quantum dots are nanometer-sized crystals of semiconducting material that provide discrete, quantized, energy levels and have a unique emission and absorbance spectrum. Quantum dot arrays deposited directly on individual pixels enable the measurement of high-resolution spectra directly by providing a unique response to the incoming light. Quantum dot spectrometers eliminate the need for optical elements such as gratings and prisms, significantly reducing cost and volume and enabling constellations of small spacecraft for observing auroral emission. Because quantum dot spectrometers can be effectively planar, the technology is also an excellent candidate for a payload on a solar sail. Solar sails offer the promise of very low-cost, propellant-less transportation across the universe, but are unable to carry significant additional mass. Integrating a quantum dot spectrometer directly into the solar sail could create a low-cost way to study the outer planets as shown in Fig. 20 [54].

### Biological and physical sciences

In addition to the Cold Atom Lab described in Sect. 2, NASA is upgrading its capabilities for in-space fundamental physics experiments through the following developments.

*Bose Einstein Condensates and Cold Atoms Lab (BECCAL):* BECCAL is a collaborative effort between NASA and DLR, the German Space Agency. BECCAL hardware is currently being built by DLR and is expected to be launched by NASA in 2027.

*The Atomic Clock Ensemble in Space (ACES):* ACES is an ESA program to launch a Cesium clock ensemble to the International Space Station in early 2025. ACES will be used for fundamental physics research, including tests of general relativity and searches for dark matter. Both NASA and NIST are collaborating in this effort and operating ground stations for time transfer.

*The Space Entanglement and Annealing Quantum Experiment (SEAQUE):* SEAQUE is currently onboard the Space Station as a technology demonstration for a photon entanglement source, and laser annealing of the photon detector to determine if high sensitivity detection can still be achieved after radiation-induced damage. This technology may be used for future fundamental physics experiments that would use quantum sensors including, for example, a mission concept like the Deep Space Quantum Link, which would, among other things, probe entanglement over large gravitational redshifts [55].

### Cross-cutting technology investments

Many sensing technologies have application across several of the science disciplines listed above. Sensor subsystems such as lasers and novel processing techniques cut across many applications and are covered here. The use of squeezed light has enabled sensing beyond the quantum limit for many applications [56], most notably the Laser Interferometer Gravitational-wave Observatory (LIGO) that detected the merging of black holes for the first time [57]. NASA is funding several small companies to develop squeezed light sources, entangled photon sources, and narrow linewidth lasers with sufficiently low power consumption, low mass and volume, and enough robustness to enable their use on spacecraft-based instruments and sensors. These efforts include:

*III/V nonlinear sources for entanglement and squeezing:* Nexus Photonics, LLC. is developing a low power, low mass, squeezed light source that packages a light squeezer and pump laser on a single chip to improve sensor sensitivity/resolution beyond the classical limits.

*IR squeezed light source:* AdvR, Inc. is developing a waveguide-based integrated source of squeezed light in the infrared for quantum sensing applications including transition edge sensors.

*Quantum light source:* Nanohmics, Inc. is developing a single-photon source that can be fabricated directly onto chip-scale devices; specifically, a near-infrared room-temperature-operational entangled photon pair source based on van der Waals ultra-thin niobium oxyhalide crystals.

*Ultra-narrow linewidth lasers:* Vescent Photonics, LLC is developing a compact, ultra-narrow linewidth laser based on photonic integrated chip technology for a trapped Sr ion clock, containing the entire laser system on a chip-scale device. This design will help reduce the size, weight, power, cost, and environmental sensitivity of high-performance clocks for deployment on space-based applications.

In addition, NASA is funding new ways to manipulate atoms promise to improve the resolution of gravity gradient measurements beyond the QGG technique described in Sect. 3.2, including the following effort:

*Optical lattice interferometer:* Led by the University of Texas at Austin, the Quantum Pathways Institute is developing a new multi-axis, optical lattice atom interferometer in a compact package by using phase-control of a 3D optical lattice structure to manipulate a laser-cooled atomic cloud. Photonic integrated circuits are being developed to guide and magnetically trap Rb atoms and to miniaturize several other optical components. The focus is on measuring gravity gradients from space with part-per-billion resolution, using a systems engineering approach to tie the instrument design to practical use on a spacecraft.

Furthermore, the Agency is sponsoring novel ways to remotely sense chemical composition that are applicable to Earth science, planetary science (for both orbital and surface systems), and astronaut health:

*Quantum-entangled spectrometer:* Physical Sciences, Inc. is developing a quantum-enhanced spectroscopic technique called “ghost spectroscopy,” which combines highly non-degenerate (different wavelength) entangled pairs of photons to probe a gas sample using infrared photons while detecting and reading out the result in the visible spectrum. This technique offers a way to probe gases without requiring high-mass cryogenic coolers for the detectors or using high-power lasers to overcome high background noise and low sensitivity.

*Neuromorphic quantum-enhanced gas sensors:* Pennsylvania State University is developing nano-engineered sensors that improve gas detection sensitivity, selectivity, and specificity while drastically lowering size, weight, and power (SWaP) through use of functionalized quantum dots and quantum materials, neuromorphic processing, and a non-traditional, new chemi-transistor device architecture.

## Possible future directions for NASA quantum sensing

Quantum sensors operating on spacecraft open entirely new regimes for Earth, planetary, and space science. For *fundamental physics*, the longer interrogation times available in a microgravity environment enable possibilities for atom interferometers to test the equivalence principle using quantum test masses, and to search for direct evidence of dark sector physics. Optical clocks, for example, could be used to look for ultralight dark matter or utilize larger gravitational baselines to enable new tests of the interplay between quantum mechanics and general relativity.

Continued advancements of technologies pertaining to the quantum components and sensors described in Sects. 2 and 3 will enable future *astrophysics* missions such as the detection of gravitational waves, dark matter and energy, and cosmic microwave background; exoplanet imaging; and detection of X-ray and far-IR emission from weak sources, photon starved regions, and from sources that require very high angular and or spectral resolution. Attributes of the sensors and components needed for these future missions are described in the 2024 NASA Astrophysics Biennial Technology Report [58].

A promising application of quantum sensing to astrophysics is the achievement of spatial super-resolution without an increase in the telescope aperture size. This goal could be achieved by breaking the diffraction limit (∼*λ*/D) through novel quantum spatial mode demultiplexing filters [59, 60]. Joint funding of this application by several government agencies has become a priority due to the potential impact on astrophysical instrumentation and potential applications on remote sensing [61]. Similarly, the detection of exoplanets could be enhanced by taking advantage of quantum state discrimination and quantum imaging techniques to reduce the probability of error for detecting planets that are at small angular separations [62]. Another promising application is the development of high-precision, quantum-based, correlated photon metrology systems that perform absolute calibration on photon detectors [63].

Additional astrophysical applications include quantum-enhanced telescopes that leverage entanglement between two telescope sites to break classical physics limits like the diffraction barrier via photon entanglement and squeezing [64]. Some of these experimental demonstrations will require entanglement-assisted interferometry, quantum memories, memory banks and spatial mode sorters [65]—capabilities that need to be developed and implemented. Further science applications include gravitation wave detection, which is the goal of the European Space Agency’s upcoming Laser Interferometer Space Antenna (LISA) mission. NASA missions such as Fermi, Swift, Hubble Space Telescope, and the James Webb Space Telescope are also being tasked to identify electromagnetic counterparts of LIGO detections. Looking beyond LISA, future programs would benefit from atom interferometry and very precise clocks. A femtosecond laser comb could be used to generate a clock signal from the transmitter, effectively suppressing laser frequency noise and clock noise for time-delay interferometry [66]. Atomic clocks with precisions of 1 x 10^−18^ placed far beyond low Earth orbit could be used to test many fundamental properties of matter, include potential variations in physical constants and providing evidence for various theories of dark matter and dark energy. Similar leaps in the performance of atom interferometers could also contribute to fundamentally improved knowledge of how matter and energy behave [67].

*Heliophysics* missions will also benefit from increased use of quantum sensors. Understanding the dynamics of hot plasmas in the solar corona is key to understanding the heating mechanisms of the solar corona and the origin of super-heated plasmas on the Sun. Just as TES sensors have provided improved performance for Astrophysics missions as described in Sect. 2, arrays of TESs could provide imaging spectroscopy to constrain the dynamics of small heating events on the Sun. Quantum magnetometers can provide increased sensitivity together with self-calibration and robust performance in the noisy electromagnetic environment of a spacecraft [68].

Measurements for future *planetary and Earth science* missions will continue to require increased spatial, temporal, and spectral resolution to reveal insights about planetary atmospheres, interiors, and surface dynamics. Quantum sensors that can fit on spacecraft with limited power, mass, and volume allocations will be critical to advancing Earth and planetary science.

A final comment on the future of quantum sensors for space science: Every quantum sensor system deployed by NASA so far, and described in Sect. 2, uses isolated quantum devices or localized quantized measurements. Entangled sensors offer opportunities that NASA has yet to take advantage of. Networks of entangled photons have already been created for terrestrial applications, including in the Chicago [69] and Washington, District of Columbia [70] regions. NASA has demonstrated round-trip Earth-to-space optical communications [71] and so could employ the same quantum entanglement protocols used by the terrestrial networks. Developing sensors based on quantum entanglement, and connecting those sensors through space-based entangled networks holds the promise of dramatically increasing the signal-to-noise ratio for each measurement, enabling, for instance, much higher resolution dark-matter searches [72] or very long-baseline interferometers formed by widely distributed spacecraft.

## Summary

The U.S. government and international organizations have made substantial investments to advance the development of quantum technologies. Many of these show great promise for disrupting sensing capabilities and NASA is at the forefront of integrating these advancements into spaceborne applications to study the nature of the universe and our place in it. NASA has a long history of using quantum sensors for astronomical and fundamental physics measurements, and continues to make advancements, primarily in reducing the size and complexity of these sensors while retaining excellent performance. While these instruments are based on quantum mechanical and electrical properties, to date all of the quantum sensing systems that NASA has deployed and is developing use isolated and highly localized measurements. Future use of networked-entangled sensors may lead to measurements that are not currently possible, leading to dramatic improvements in our ability to understand the Universe.

## Data Availability

No datasets were generated or analysed during the current study.
